# Numerical study on a rotational hydraulic damper with variable damping coefficient

**DOI:** 10.1038/s41598-021-01859-2

**Published:** 2021-11-18

**Authors:** Huiyong Zhao, Baohua Wang, Genfu Chen

**Affiliations:** School of Automotive Engineering, Hubei University of Automotive Technology, Shiyan, China

**Keywords:** Engineering, Mechanical engineering

## Abstract

The rotational hydraulic damper has advantages in the design and control of rotational machines. This paper presents a novel hydraulic rotational damper with the characteristic of adjusting the damping coefficient. It is composed of a shell, a gap, a rotor shaft, sliding vanes, a valve, and a motor, just like a combination of a sliding pump system and a valve driven by a motor. A new cam ring slot designed to guide the radial motion of sliding vanes could reduce friction resistance force, which will also benefit the design of the sliding pump. The damping coefficient model of this damper is established based on dynamic analysis. Series of numerical simulations validate the impact of factors on the damping coefficient. Frictional resistances have little influence on the damping coefficient during most conditions. The total coefficient is positively correlative with the angular velocity and the valve angle. Therefore, changing the valve angle according to the rotor shaft’s angular speed could adjust the damping coefficient.

## Introduction

The vibration, especially nonlinear vibration, seriously harms the operation the mechanical system, which will result in the chaos and bifurcation of the system^1^. The damper is an essential system to eliminate the vibration of the mechanical systems. It has three basic structural types, cylindrical, rotational and the tuned mass damper, and three control modes, passive, active, semi-active. Researchers developed various dampers based on these structural types and control modes. The cylindrical damper is common in many areas, i.e., automotive engineering, structural engineering. Many kinds of cylinder dampers are adopted to improve the performance of the automotive suspension^2–4^ or achieve better structural control of tall buildings^5–7^. The application of the second concentrates on the region of structural engineering^8–10^. Some researchers designed rotational friction dampers to enhance the seismic performance of inelastic structures^11–14^. Others developed an energy dissipation outrigger system with a rotational inertia damper^9,15^. However, in the automotive engineering area, similar literals are very few.

In the vehicle systems, the working mode of many assemblies are similar to that of a rotational damper. As is known, the torque coupler, i.e., clutch, synchronizer, or brake, is essential in the automotive chassis system^16^. Its transmitted torque increases linearly with the pressure force acting on the friction surface while the angular speed difference between the input and output shafts decreased to zero, just like one kind of variable coefficient rotational friction damper. The torsional vibration isolator equipped in automotive clutches and engines is another typical assembly. It works in the friction mode^17^, also like a rotational friction damper. A long time of working will wear the contact parts and may lead to the cost and reliability problems of the whole system. A variable damping coefficient hydraulic rotational damper may be a better alternative. Magnetorheological fluid rotational dampers (MR rotational dampers) have excellent performance due to the viscosity of MR fluid controlled by the strength of the magnetic field^18^. But their rotational angle is less than 360°^19,20^. So, it is significant to develop a new hydraulic rotational damper that could change the coefficient in a wide range with an infinite rotational angle.

Hydraulic dampers produce resistance torques or forces by letting the fluid passes through the orifice^21,22^. The torques or forces are positively correlative to the cross-sectional area of the orifice and the velocity of the fluid^23,24^. Thus, the rotational hydraulic damper is like a rotational pump with a valve. With this idea, some authors proposed a concept of an energy-saving steering system and detailed its control scheme^25^. The sliding vane pump is a popular rotational pump. Researchers developed its new structures^26,27^, established its dynamic model, and discussed its mechanical and fluid characteristics^28–32^. Besides, researchers concentrate on the orifice's characteristics based on the tube hydraulic dampers^1,33–35^. Some acquire the ball valve's local fluid pressure dropping characteristic curves with mathematical calculation, CFD simulation, and experiment^36,37^. Although these literals benefit in the design of the rotational hydraulic damper, the cam ring slot of those sliding vane pumps is unsuitable. The periodically continuous varying of the pressure angle in this cam ring slot makes the sliding vane suffer pressure drop during the radial motion. It increases the friction resistance and the worn of sliding vanes and decreases the efficiency of the system. However, studies on them are very few to the extent of the authors' ability.

In this paper, we proposed a scheme of a new variable damping coefficient rotational hydraulic damper, which is a semi-active rotational damper. The factors that influenced the damping coefficient are discussed in detail with simulation. The outcomes will benefit a lot to the structure and performance improvement of this damper and vane pumps. The remains of this paper are organized as follows. In “Concept of the system”, the structure and working principle of the damper are outlined. In “Design of the cam ring”, the model of the new cam ring curve is deduced and discussed. In “Coefficient model of the damper”, the damper’s dynamics model is established, and its coefficient model of the damper is derived. The concluding remarks are given in “Simulation and analysis”.

## Concept of the system

**Figure 1:**
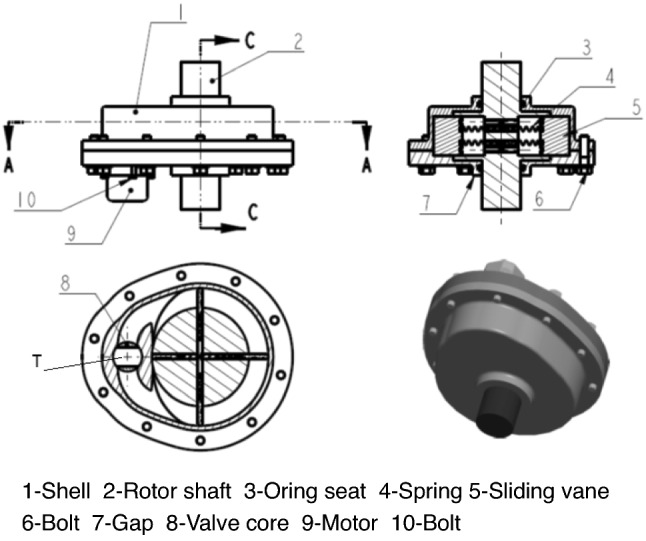
Structure of the rotational hydraulic damper. (Creo/parametric, trial version. www.ptc.com/products/creo/).

As shown in Fig. 1 (drawn with the software Creo/parametric, trial version. Available from www.ptc.com/products/creo/), the proposed damper is composed of a rotating rotor, also named as center shaft, with grooves hosting four parallelepiped vanes, a concentrically mounted cam ring, a valve core, two symmetric working chambers, a shell and a gap, springs and seal rings, and a motor^38^. The inner cavity of the damper is full of fluid. As the rotor rotates, the dynamic behavior of sliding motion combined with rotation and spring forces leads the vanes to contact the inner surface of the cam ring. Thus, the vanes pushed the fluid to pass through tunnel T, which produced the fluid pressure difference between the two sides of the tunnel. This pressure difference acts on the vanes and produces a resistant torque. At the same time, the motion of the sliding vanes and the rotating of the rotor shaft generates frictional resistance moments.

The damper coefficient is the quotient of the total resistance torque and the velocity of the rotor shaft. The resistance torque is the sum of the friction torques and the torque produced by the pressure drop acting on the sliding vanes. So, the damping coefficient is the sum of the two coefficients. The first is the frictional damping coefficient, deduced with a dynamic analysis of the vanes and the rotor shaft. The latter is the damping coefficient caused by fluid resistance, mainly influenced by the shape of the tunnel and calculated with the fluid analysis of the damper.

**Figure 2:**
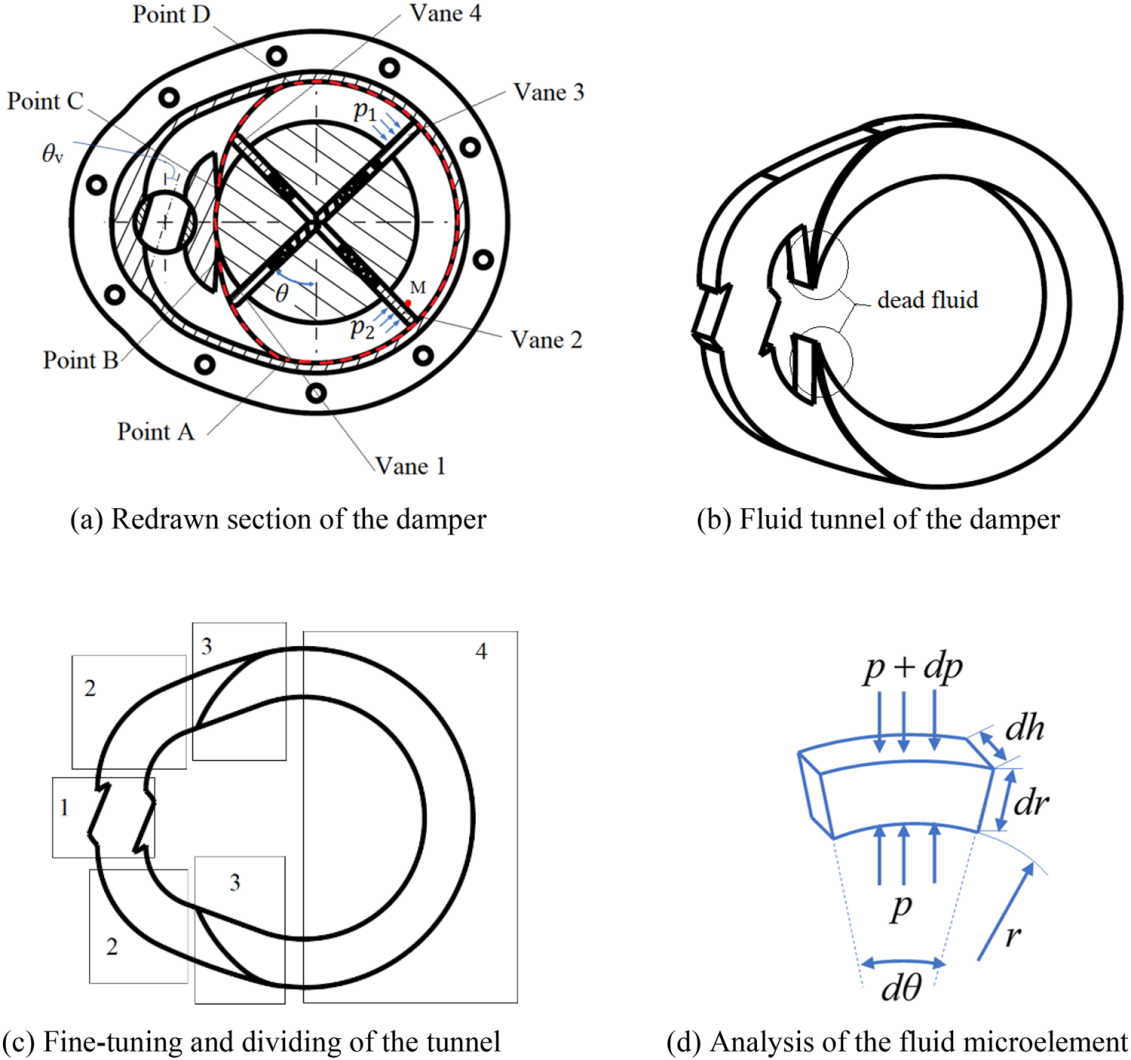
Fluid tunnel and microelement analysis of the damper. (Figures (**a**)–(**c**) are drawn with the software Creo/parametric, trial version. Figure (**d**) is drawn with the drawing tools of the Microsoft Word software and saved as a picture).

To analyze the fluid resistance in detail, the section view of the damper is redrawn, as shown in Fig. 2a. Here, the rotation angle of the valve is denoted as $\theta_{{\text{V}}}$, the fluid pressure in the two working chambers is denoted as $p_{1}$ and $p_{2}$, respectively, and the rotational angle of the rotor is denoted as $\theta$. The red dashed line is the slot of the cam ring, which is composed of two arcs and two transition curves and will be detailed in “Design of the cam ring”. Point A and C are the start points of the transition curves, point B and D are the endpoints of the transition curves. The fluid tunnel is extracted as shown in Fig. 2b and divided into four parts as shown in Fig. 2c. The fluid microelement in the circular part of the tunnel is selected to analyze the pressure variation in the radial direction.

There are two horn parts in the whole extracted tunnel of the fluid, as is shown in Fig. 2b. If ignoring the impact of the fluid vortex, these two parts will not participate in the fluid recycle and could be defined as dead fluid. Thus, the tunnel could be simplified to a three-dimensional model composed of six parts, as shown in Fig. 2c. Part 1 is the fluid tunnel of the cylindrical ball valve, whose section could be changed by rotating the valve with the motor. Part 2 and part 3 is the smooth-walled bend and the sudden expansion–contraction tunnel, respectively. And part 4 is the circular part of the tunnel.

The first three parts are the main components to produce the local resistance of the fluid. For part 2 and part 3, their shapes are unchanged after being manufactured. Therefore, their local resistance coefficients are constant. The section of part 1 varies with the rotating the valve core, and its local resistance coefficient is a function of the angle of the valve. Reference^38^ presents the method to deduce the expressions of these local resistance coefficients. So, in this paper, we utilize the function $f{(}\theta_{{\text{V}}} {)}$ to describe the total local resistance coefficient. The range of $\theta_{{\text{V}}}$ is from zero to a constant less than 90°. The fluid resistance and the damping coefficient are small when $\theta_{{\text{V}}}$ is in the maximum value. Similarly, fluid resistance will be very high when $\theta_{{\text{V}}}$ is zero due to the incompressibility of fluids. So, $f{(}\theta_{{\text{V}}} {)}$ is a monotone increasing function.

The fluid motion is a forced vortex in part 4, which will produce additional pressure. To simplify the analysis, the rotational velocity of the rotor shaft, $\dot{\theta }$, is assumed to be constant. Pick a fluid element in part 4 near the point D as a control volume, as shown in Fig. 2d, where $p$ is pressure acting on the inner side of the control volume, $p + dp$ is pressure acting on the outer side of the control volume, $dh$ and $dr$ are the section sizes of the control volume, $r$ is the distance from the axis of the shaft to the inner side of the control volume. And Eqs. (1), (2) is deduced with newton’s law.

1 $${(}p_{1} + dp_{1} {)}r\;dh\;d\theta - p_{1} r\;dh\;d{\kern 1pt} \theta = \rho r\dot{\theta }^{2} dr\;dh\;d\theta .$$

2 $$p_{1} = \rho r\dot{\theta }^{2} + c_{1} ,$$

where $\rho$ is the density of the fluid, $c_{1}$ is a constant. Similarly, $p_{2}$ and the pressure drop are easily deduced and shown in Eq. (3). The pressure drop is not related to the rotational speed of the rotor shaft, as shown in Eq. (4).

3 $$p_{2} = \rho r\dot{\theta }^{2} + c_{2} .$$

4 $$\Delta p = p_{2} - p_{1} = c_{2} - c_{1} .$$

This pressure drop is the local fluid resistance, is the function of the two variables, fluid velocity and fluid resistance coefficient of the damper^39^. Define the average velocity of the fluid in part 4 as $v$, and pressure drop is,

5 $$\Delta p = p_{2} - p_{1} = f{(}\theta_{{\text{v}}} {)}\rho \frac{{v^{2} }}{2}.$$

Therefore, the fluid in the tunnel could be divided into three parts: high-pressure area, low-pressure area, and neutral area. The regions of the high-pressure and low-pressure areas vary along with the moving of the vanes. When vane 1 moves along the slot curve from point A to Point B, vane 4 will be along the slot curve from Point C to Point D, while vane 2 and vane 3 still move along the circular part of the slot from Point D to Point A. Thus, vane 2 and vane 3 will be components to compose the high-pressure area and low-pressure area, respectively. The area in the circular part of the tunnel from vane 2 to vane 3 is defined as a neutral area. If the end surface of vane 2 passes through Point A, vane 3 and vane 4 will be used as alternatives to form the high-pressure area, low-pressure area, and neutral area. In the neutral and the low-pressure areas, the liquid is not compressed. The liquid pressure is assumed the same.

## Design of the cam ring

In this section, a geometric model of the novel cam ring profile is established, which is composed of two concentric arc curves and two transition curves. The dynamic characteristics of the proposed cam ring profiles are analyzed.

### Modelling of the transition curve

**Figure 3:**
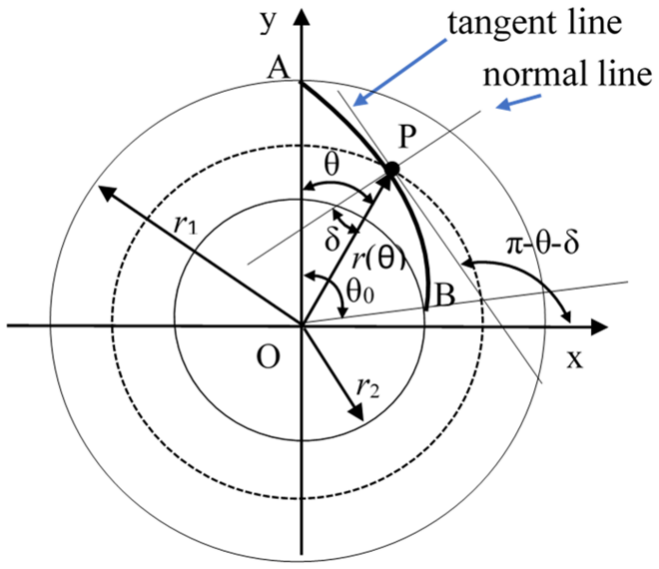
Geometric relationships of the transition curve. (Figure is drawn with the drawing tools of the Microsoft Word software and saved as a picture).

Transition curves are connecting curves between two concentric arcs with different radius. It is a guide curve to drive the sliding vanes moving in the radial direction smoothly. Figure 3 describes a transition curve from the bigger circle to a smaller concentric one. In this figure, P is the contact point between the sliding vane and the cam slot in the bottom view, Point A and Point B is the start point and the endpoint of the transition curve, respectively, Point O is the center of the concentric circle, $r_{1}$ is the radius of the bigger circle which is also the radius of the inner surface of the shell, $\delta$ is the angle between the line OP and the normal direction of the transition curve at point P, line OA is in coincide with the y-axis of the Cartesian coordinate system, $\theta$ is the angle between the line OP and y-axis, $r{(}\theta { )}$ is the length of the line OP, $\theta_{0}$ is the angle between the line OA and OB.

Define the coordinate of point P as $(x,y)$, the transition curve could be described as,

6 $$x=r\left(\theta \right)\cdot \mathrm{sin}\left(\theta \right),$$

7 $$y=r\left(\theta \right)\cdot \mathrm{cos}\left(\theta \right).$$

The slope of the tangent line of the transition curve at point P is,

8 $$K = \frac{dy}{{dx}} = \frac{{\dot{r}{(}\theta {)}\;{\text{cos(}}\theta {)} - r{(}\theta {)}\;{\text{sin(}}\theta {)}}}{{\dot{r}{(}\theta {)}\;{\text{sin(}}\theta {)} + r{(}\theta {)}\;{\text{cos(}}\theta {)}}}.$$

It is easily deduced from the relationship of trigonometric geometry that $\pi - \theta - \delta$ is the angle between this tangent line and the x-axis. So, there is,

9 $$\frac{{\dot{r}(\theta {)}\;{\text{cos(}}\theta {)} - r{(}\theta {)}\;{\text{sin(}}\theta {)}}}{{\dot{r}(\theta {)}\;{\text{sin(}}\theta {)} + r{(}\theta {)}\;{\text{cos(}}\theta {)}}} = {\text{tan}}\;{(}\pi - \theta - \delta {)}$$

and

10 $$r{(}\theta {)} = r_{1} e^{{ - \int_{0}^{\theta } {{\text{tan(}}\theta {)}d\theta } }} .$$

Similarly, the transition curve from the smaller circle to a bigger one is also deduced, as shown in Eq. (11).

11 $$r{(}\theta {)} = r_{2} e^{{\int_{0}^{\theta } {{\text{tan(}}\theta {)}d\theta } }} .$$

### The novel cam ring profile

Denote the central angle of the smaller arc as $\theta_{s0}$, the whole cam ring profile could be described with a piecewise function in polar coordinates, as shown in follows,

12 $$r(\theta ) = \left\{ {\begin{array}{*{20}l} {r_{1} e^{{ - \int_{0}^{\theta } {{\text{tan(}}\delta {)}d\theta } }} } \hfill & {0 \le \theta < \theta_{0} } \hfill \\ {r_{2} } \hfill & {\theta_{0} \le \theta < \theta_{0} + \theta_{s0} } \hfill \\ {r_{2} e^{{{\int_{0}{\theta - \theta_{0} - \theta_{s0} }} {{\text{tan(}}\delta {)}d\theta } }} } \hfill & {\theta_{0} + \theta_{s0} \le \theta < 2\theta_{0} + \theta_{s0} } \hfill \\ {r_{1} } \hfill & {2\theta_{0} + \theta_{s0} \le \theta < 2\pi } \hfill \\ \end{array} } \right..$$

The radial motion of the sliding vane generates a frictional resistance force between the sliding vane and its groove. This force grows with the increase of the pressure angle, which may lead to the self-locking between sliding vanes and the groove. To avoid this, the value of the pressure angle should be less than the maximum value $\delta_{{{\text{max}}}}$. Assume the friction coefficient is $\mu$, and the maximum value of the pressure angle is,

13 $$\delta_{{{\text{max}}}} = {\text{arctan(}}{1 \mathord{\left/ {\vphantom {1 \mu }} \right. \kern-\nulldelimiterspace} \mu }{)}{\text{.}}$$

To eliminate the shock vibration caused by the sudden change of the sliding vane’s radial velocity, the pressure angle $\delta$ is designed as a piecewise continuous function, as shown in Eq. (14)

14 $$\delta = \left\{ {\begin{array}{*{20}l} {g{(}\theta {)}} \hfill & {0 \le \theta < {{\theta_{0} } \mathord{\left/ {\vphantom {{\theta_{0} } k}} \right. \kern-\nulldelimiterspace} k}} \hfill \\ {\delta_{0} } \hfill & \begin{gathered} {{\theta_{0} } \mathord{\left/ {\vphantom {{\theta_{0} } k}} \right. \kern-\nulldelimiterspace} k} \le \theta < \theta_{0} - {{\theta_{{{\text{s}}0}} } \mathord{\left/ {\vphantom {{\theta_{{{\text{s}}0}} } k}} \right. \kern-\nulldelimiterspace} k} \hfill \\ {\text{or}}\;\theta_{0} + \theta_{s0} + {{\theta_{0} } \mathord{\left/ {\vphantom {{\theta_{0} } k}} \right. \kern-\nulldelimiterspace} k} \le \theta < 2\theta_{0} + \theta_{s0} - {{\theta_{0} } \mathord{\left/ {\vphantom {{\theta_{0} } k}} \right. \kern-\nulldelimiterspace} k} \hfill \\ \end{gathered} \hfill \\ {g{(}\theta_{0} - \theta {)}} \hfill & {\theta_{0} - {{\theta_{0} } \mathord{\left/ {\vphantom {{\theta_{0} } k}} \right. \kern-\nulldelimiterspace} k} \le \theta < \theta_{0} } \hfill \\ {g{(}\theta - \theta_{0} - \theta_{s0} {)}} \hfill & {\theta_{0} + \theta_{s0} \le \theta < \theta_{0} + \theta_{s0} + {{\theta_{0} } \mathord{\left/ {\vphantom {{\theta_{0} } k}} \right. \kern-\nulldelimiterspace} k}} \hfill \\ {g{(2}\theta_{0} + \theta_{s0} - \theta {)}} \hfill & {2\theta_{0} + \theta_{s0} - {{\theta_{0} } \mathord{\left/ {\vphantom {{\theta_{0} } k}} \right. \kern-\nulldelimiterspace} k} \le \theta < 2\theta_{0} + \theta_{s0} } \hfill \\ 0 \hfill & {\theta_{0} \le \theta < \theta_{0} + \theta_{s0} \;{\text{or}}\;2\theta_{0} + \theta_{s0} \le \theta < 2\pi } \hfill \\ \end{array} } \right.,$$

where, $k$ is a proportional coefficient, $\delta_{0}$ is the specified value of the pressure angle, $\delta_{0} < \delta_{{{\text{max}}}}$, $g{(}\theta {)}\;$ is a monotonically increasing function with the initial and final condition of $g{(}0{)}\; = 0$ and $g{(}{{\theta_{0} } \mathord{\left/ {\vphantom {{\theta_{0} } k}} \right. \kern-\nulldelimiterspace} k}{)}\; = \delta_{0}$, respectively. Given the specific expression of $g{(}\theta {)}\;$, every parameter could be calculated once specifying values of rest parameters, and the cam ring slot could be drawn with Matlab software.

## Coefficient model of the damper

In this section, the mathematical model of the damping coefficient is developed based on the dynamic model of the damper. The dynamic model is composed of the motions of the sliding vanes and the rotating rotor. However, the dynamic characteristics and the control strategy of the valve core are neglected in the damping coefficient modeling because forces and torques exerted on the valve do not influence the damping coefficient model.

### Dynamic model of sliding vanes

**Figure 4:**
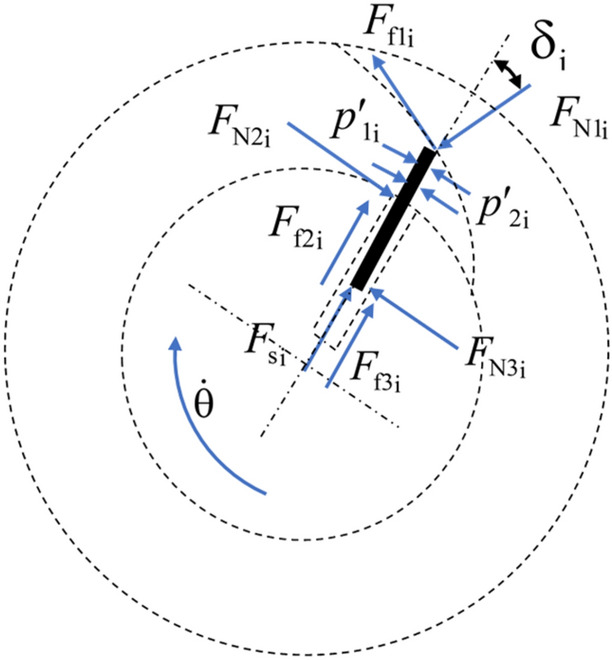
Schematic diagram of the motion of sliding vane $i$ (Fig. 3 is drawn with the drawing tools of the Microsoft Word software and saved as a picture).

The dynamic analysis of the sliding vanes is a prerequisite in the modeling of the damping coefficient. Sliding vanes have two states, having or having no radial motion. The first occurs when moving along with circular curves. The second occurs when moving along with transition curves. The sliding vanes suffer two more resistance forces in the second state when defining the contacts among the groove, the cam ring slot, and the sliding vane as point contact, as shown in Fig. 4. $F_{{{\text{f}}2{\text{i}}}}$, $F_{{{\text{f}}3{\text{i}}}}$ and $F_{{{\text{f}}1{\text{i}}}}$ denote the frictional resistance forces rotor groove and cam ring acting on the contact point of the sliding vane ${\text{i}}$, respectively. $F_{{{\text{N}}2{\text{i}}}}$, $F_{{{\text{N}}3{\text{i}}}}$ and $F_{{{\text{N}}1{\text{i}}}}$ are the normal forces that the rotor groove and cam ring acting on the sliding vane ${\text{i}}$, respectively. $F_{{s{\text{i}}}}$ is the spring force acting on the sliding vane ${\text{i}}$. $p^{\prime}_{{1{\text{i}}}}$ and $p^{\prime}_{{2{\text{i}}}}$ denote the fluid pressure acting on each side of the vane ${\text{i}}$. If $\delta_{{\text{i}}}$ is zero, the sliding vane will have no radial motion, values of the two additional frictional resistances, $F_{{{\text{f}}2{\text{i}}}}$ and $F_{{{\text{f}}3{\text{i}}}}$, are zero. In ideal status, $p^{\prime}_{{1{\text{i}}}}$ equals $p^{\prime}_{{2{\text{i}}}}$ when the sliding vane ${\text{i}}$ is in the low-pressure area or along the transition curves. If the sliding vane ${\text{i}}$ is the boundary between the high-pressure area and low-pressure area, $p^{\prime}_{{1{\text{i}}}}$ and $p^{\prime}_{{2{\text{i}}}}$ equal $p_{1}$ and $p_{2}$, respectively. The vane's mass and height are denoted as $m$ and $h$, respectively, and $l_{s}$ is the length of the sliding vane. So, the following equations are deduced based on the force and torque equilibrium laws and could represent the whole general model of the damper.

15 $$F_{{{\text{si}}}} + F_{{{\text{f}}2i}} + F_{{{\text{f}}3i}} - F_{{{\text{N1i}}}} {\text{cos(}}\delta_{{\text{i}}} {)} + F_{{{\text{f}}1{\text{i}}}} {\text{sin(}}\delta_{{\text{i}}} {)} + {{m{(}r{(}\theta_{{\text{i}}} {)} + r_{2} {)}\dot{\theta }^{2} } \mathord{\left/ {\vphantom {{m{(}r{(}\theta_{{\text{i}}} {)} + r_{2} {)}\dot{\theta }^{2} } 2}} \right. \kern-\nulldelimiterspace} 2} = m\ddot{r}{(}\theta_{{\text{i}}} {)}{\text{.}}$$

16 $$F_{{{\text{N1i}}}} {\text{sin(}}\delta_{{\text{i}}} {)} + F_{{{\text{N3i}}}} - F_{{{\text{N2i}}}} + F_{{{\text{f}}1{\text{i}}}} {\text{cos(}}\delta_{{\text{i}}} {)} + {(}r{(}\theta_{{\text{i}}} {)} - r_{2} {)}h{(}p_{{2{\text{i}}}}^{\prime } - p_{{1{\text{i}}}}^{\prime } {)} = m{(}r{(}\theta_{{\text{i}}} {)} - {{l_{s} } \mathord{\left/ {\vphantom {{l_{s} } {2{)}}}} \right. \kern-\nulldelimiterspace} {2{)}}}\ddot{\theta }.$$

17 $$\begin{aligned} & {(}F_{{{\text{N1i}}}} {\text{sin(}}\delta_{{\text{i}}} {)} + F_{{{\text{f}}1{\text{i}}}} {\text{cos(}}\delta_{{\text{i}}} {))(}r{(}\theta_{{\text{i}}} {)} - r_{2} {)} - F_{{{\text{N3i}}}} {(}l_{s} - r{(}\theta_{{\text{i}}} {)} + r_{2} {)} \\ & \quad + {{{(}r{(}\theta_{{\text{i}}} {)} - r_{2} {)}^{2} h{(}p_{{2{\text{i}}}}^{\prime } - p_{{1{\text{i}}}}^{\prime } {)}} \mathord{\left/ {\vphantom {{{(}r{(}\theta_{{\text{i}}} {)} - r_{2} {)}^{2} h{(}p_{{2{\text{i}}}}^{\prime } - p_{{1{\text{i}}}}^{\prime } {)}} 2}} \right. \kern-\nulldelimiterspace} 2} = m{(}r{(}\theta_{{\text{i}}} {)} - {{l_{s} } \mathord{\left/ {\vphantom {{l_{s} } {2{)}}}} \right. \kern-\nulldelimiterspace} {2{)}}}{(}r_{2} - r{(}\theta_{{\text{i}}} {)} + {{l_{s} } \mathord{\left/ {\vphantom {{l_{s} } {2{)}}}} \right. \kern-\nulldelimiterspace} {2{)}}}\ddot{\theta }. \\ \end{aligned}$$

18 $$F_{{{\text{f}}1{\text{i}}}} = F_{{{\text{N1i}}}} \mu .$$

19 $$F_{{{\text{f2i}}}} = \left| {F_{{{\text{N2i}}}} } \right|\mu {\text{sign(}}\dot{r}{(}\theta_{{\text{i}}} {))}{\text{.}}$$

20 $$F_{{{\text{f3i}}}} = \left| {F_{{{\text{N3i}}}} } \right|\mu {\text{sign(}}\dot{r}{(}\theta_{{\text{i}}} {))}{\text{.}}$$

21 $$F_{{{\text{si}}}} = K_{{\text{s}}} {(}r_{1} - r{(}\theta_{{\text{i}}} {)} + l_{s} {))}{\text{.}}$$

Taking the sliding vanes and the rotor shaft as a whole system, the external force acting on the sliding vane are $F_{{{\text{f}}1{\text{i}}}}$ and $F_{{{\text{N}}1{\text{i}}}}$. Thus, the external friction torque acting on the sliding vane, $T_{{{\text{f}}1{\text{i}}}}$, is described with Eq. (22).

22 $$T_{{{\text{f}}1{\text{i}}}} = r{(}\theta_{{\text{i}}} {)(}F_{{{\text{f}}1{\text{i}}}} {\text{cos(}}\delta_{{\text{i}}} {)} + F_{{{\text{N}}1{\text{i}}}} {\text{sin(}}\delta_{{\text{i}}} {))}{\text{.}}$$

**Figure 5:**
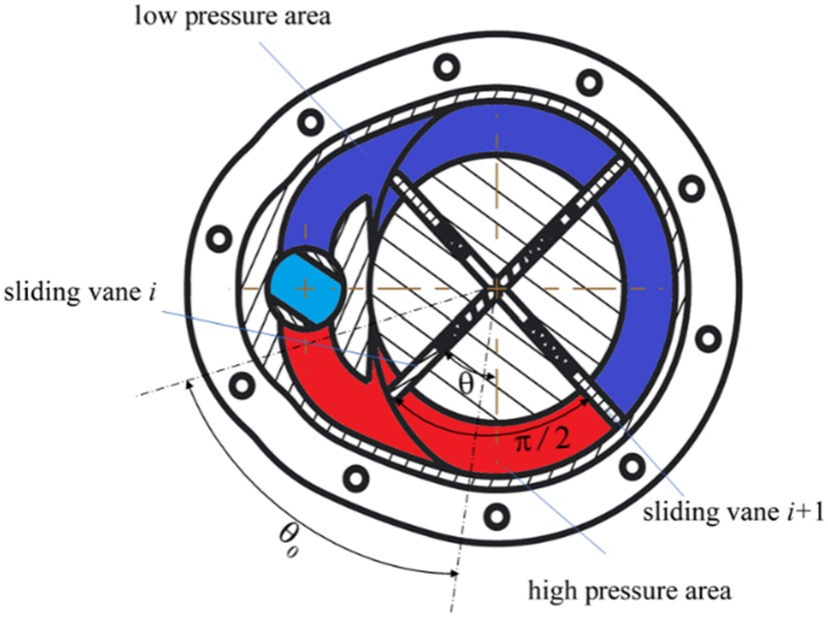
Sketch map of the fluid pressure. (Figure is created with the software Creo/parametric 2.0 and modified with the drawing software integrated in the Win 10 operation system).

The cylindrical surface of the rotor shaft also bears the normal force exerting by the fluid pressure drop, as shown in Fig. 5. The amplitude and direction of this force vary periodically due to the periodic variating of the high-pressure area of the cylinder surface. Define this force as $F_{{\text{a}}}$, there is,

23 $$F_{{\text{a}}} = 2{(}p_{2} - p_{1} {)}hr_{2} {\text{sin}}\left( {\frac{{\theta_{0} }}{{2}} - \frac{\theta }{2} + \frac{\pi }{4}} \right){.}$$

Denote the friction coefficient between the rotor and the shell as $\mu_{r}$ and the radius of the shaft’s friction surface as $r_{r}$, and the frictional torque that the shell acts on the rotor shaft is described with Eq. (24).

24 $$T_{{\text{a}}} = \left( {F_{{\text{a}}} + \sum {F_{{{\text{f2}}}} } } \right)r_{r} \mu_{r} .$$

### Dynamic models of the rotational shaft

The fluid pressures exert on the sides of the vanes and produce a resistance torque, which is the main component to block the motion of the shaft. As detailed previously, the fluid in the tunnel is divided into three parts: high-pressure area, low-pressure area, and neutral area. The fluid's pressure in the neutral area equals that in the low-pressure area. Only the vane separating connecting the neutral area and the high-pressure area suffers two different pressures. Thus, this resistance torque could be calculated with the principle of calculus, as shown in Eq. (25).

25 $$T_{{{\text{rf}}}} = \int\limits_{{r_{0} }}^{{r_{1} }} {\Delta phrdr = \frac{1}{2}\Delta ph{(}r_{1}^{2} - r_{2}^{2} {)}} {.}$$

The torque equilibrium equation of the damper is deduced, as is shown in Eq. (26)

26 $$T_{{{\text{in}}}} - T_{{{\text{rf}}}} - \sum {T_{{{\text{f1i}}}} } - T_{{\text{a}}} - T_{{{\text{out}}}} = J_{{{\text{damp}}}} \ddot{\theta }.$$

where $i$ is the number of the sliding vane, $T_{{{\text{in}}}}$ and $T_{{{\text{out}}}}$ are input torque and output torque of the rotor shaft, respectively.

### Damping coefficient

As is known, the damping torque of the rotational damper is the total resistance torque of the rotational damper, which equals the product of the angular velocity of the rotor shaft and rotational damping coefficient. For this damper, the rotational speed of the rotor shaft is utilized to calculate the damping coefficient. Because the resistance torque of the damper is the total of $\sum {T_{{{\text{f1i}}}} }$, $T_{{{\text{rf}}}}$, and $T_{{\text{a}}}$, the damping coefficient is divided into three components to discuss the influencing factors, which are vane friction damping coefficient, shaft friction damping coefficient, and fluid local resistance coefficient. The damping coefficient and its components, denoted as $B$, $B_{{{\text{a1}}}}$, $B_{{{\text{a2}}}}$ and $B_{{{\text{a3}}}}$, respectively, could be calculated with the following equations.

27 $$B = \frac{{T_{{{\text{rf}}}} + \sum {T_{{{\text{f1i}}}} } + T_{{\text{a}}} }}{{\dot{\theta }}} = B_{{{\text{a1}}}} + B_{{{\text{a2}}}} + B_{{{\text{a3}}}} .$$

28 $$B_{{{\text{a1}}}} = \frac{{\sum {T_{{{\text{f1i}}}} } }}{{\dot{\theta }}} = \frac{{\sum {(} r{(}\theta_{{\text{i}}} {)(}F_{{{\text{f}}1{\text{i}}}} {\text{cos(}}\delta_{{\text{i}}} {)} + F_{{{\text{N}}1{\text{i}}}} {\text{sin(}}\delta_{{\text{i}}} {))}}}{{\dot{\theta }}}.$$

29 $$B_{{{\text{a2}}}} = \frac{{T_{{\text{a}}} }}{{\dot{\theta }}} = \left( {f{(}\theta_{{\text{v}}} {)}\rho \frac{{{(}r_{1} + r_{2} {)}^{2} }}{4}\dot{\theta } + \frac{{\sum {F_{{{\text{f2}}}} } }}{{\dot{\theta }}}} \right)r_{r} \mu_{r} hr_{2} {\text{sin}}\left( {\frac{{\theta_{0} }}{{2}} - \frac{\theta }{2} + \frac{\pi }{4}} \right){.}$$

30 $$B_{{{\text{a3}}}} = \frac{{T_{{{\text{rf}}}} }}{{\dot{\theta }}} = f(\theta_{{\text{v}}} )\rho h\frac{{{(}r_{1} + r_{2} {)}^{2} {(}r_{1}^{2} - r_{2}^{2} {)}}}{16}\dot{\theta }.$$

It is clear from Eq. (28) to (30) that $B_{{{\text{a1}}}}$ and $B_{{{\text{a2}}}}$ periodically vary along with the increase of the rotor shaft’s angular velocity, and the amplitudes of $B_{{{\text{a2}}}}$ and $B_{{{\text{a3}}}}$ grow along with the increase of the shaft’s rotational speed and the local fluid resistance coefficient. The local fluid resistance coefficient decreases along with the increase of the valve’s angle, $\theta_{{\text{v}}}$. The amplitudes of $B_{{{\text{a2}}}}$ and $B_{{{\text{a3}}}}$ could be adjusted online by rotating the valve. Consequently, the total coefficient, $B$, is also adjustable.

## Simulation and analysis

In this section, the cam ring slot and the local fluid resistance coefficient of this rotational damper are calculated and analyzed in Matlab (Matlab2019a. Available from https://ww2.mathworks.cn/products/get-matlab.html?s_tid=gn_getml) and a commercial computational fluid dynamics (CFD) software, FloEFD (FloEFD, trial version. https://www.plm.automation.siemens.com/global/en/products/simcenter/floefd.html), with given parameters. The damping coefficient and other characteristics of the damper are simulated with Matlab/Simulink software.

### Calculation of the cam ring slot

As mentioned previously, $g{(}\theta {)}\;$ is the control function to describe the pressure angle $\delta$. Considering $\delta$ varying continuously, $g{(}\theta {)}\;$ has three constraints, as listed from Eq. (31) to Eq. (33).

31 $$g{(}0{)} = 0.$$

32 $$g{(}{{\theta_{0} } \mathord{\left/ {\vphantom {{\theta_{0} } k}} \right. \kern-\nulldelimiterspace} k}{)} = \delta_{0} .$$

33 $$r_{1} e^{{{ - \int_{0}{{{\theta_{0} } \mathord{\left/ {\vphantom {{\theta_{0} } k}} \right. \kern-\nulldelimiterspace} k}}} {{\text{tan(}}g{(}\theta {))}d\theta } }} = r_{1} e^{{{ - \int_{0}{{{\theta_{0} } \mathord{\left/ {\vphantom {{\theta_{0} } k}} \right. \kern-\nulldelimiterspace} k}}} {{\text{tan(}}\delta_{0} {)}d\theta } }} .$$

In this paper, $g{(}\theta {)}\;$ is a linear function, $k\delta_{0} {\theta \mathord{\left/ {\vphantom {\theta {\theta_{0} }}} \right. \kern-\nulldelimiterspace} {\theta_{0} }}$, and the value of the parameter $k$ is easily calculated with Eq. (34). Table 1 presents the initial value of each parameter. The three-dimension model of the damper’ prototype is drawn based on these parameters.

34 $$k = \frac{{2\theta_{0} }}{{\delta_{0} }}\frac{{{\text{ln(cos(}}\delta_{0} {))} + \delta_{{0}} {\text{tan(}}\delta_{{0}} {)}}}{{\theta_{{0}} {\text{tan(}}\delta_{{0}} {)} - {\text{ln(}}{{r_{1} } \mathord{\left/ {\vphantom {{r_{1} } {r_{2} {)}}}} \right. \kern-\nulldelimiterspace} {r_{2} {)}}}}}.$$

**Table 1:** Parameters of the cam ring slot.

Parameter	Value	Unit
$\delta_{0}$	${{17\pi } \mathord{\left/ {\vphantom {{17\pi } {180}}} \right. \kern-\nulldelimiterspace} {180}}$	rad
$\theta_{0}$	${{7\pi } \mathord{\left/ {\vphantom {{7\pi } {18}}} \right. \kern-\nulldelimiterspace} {18}}$	rad
$\theta_{{{\text{s}}0}}$	${\pi \mathord{\left/ {\vphantom {\pi 9}} \right. \kern-\nulldelimiterspace} 9}$	rad
$k$	10.2326	
$r_{1}$	70	mm
$r_{2}$	50	mm

**Figure 6:**
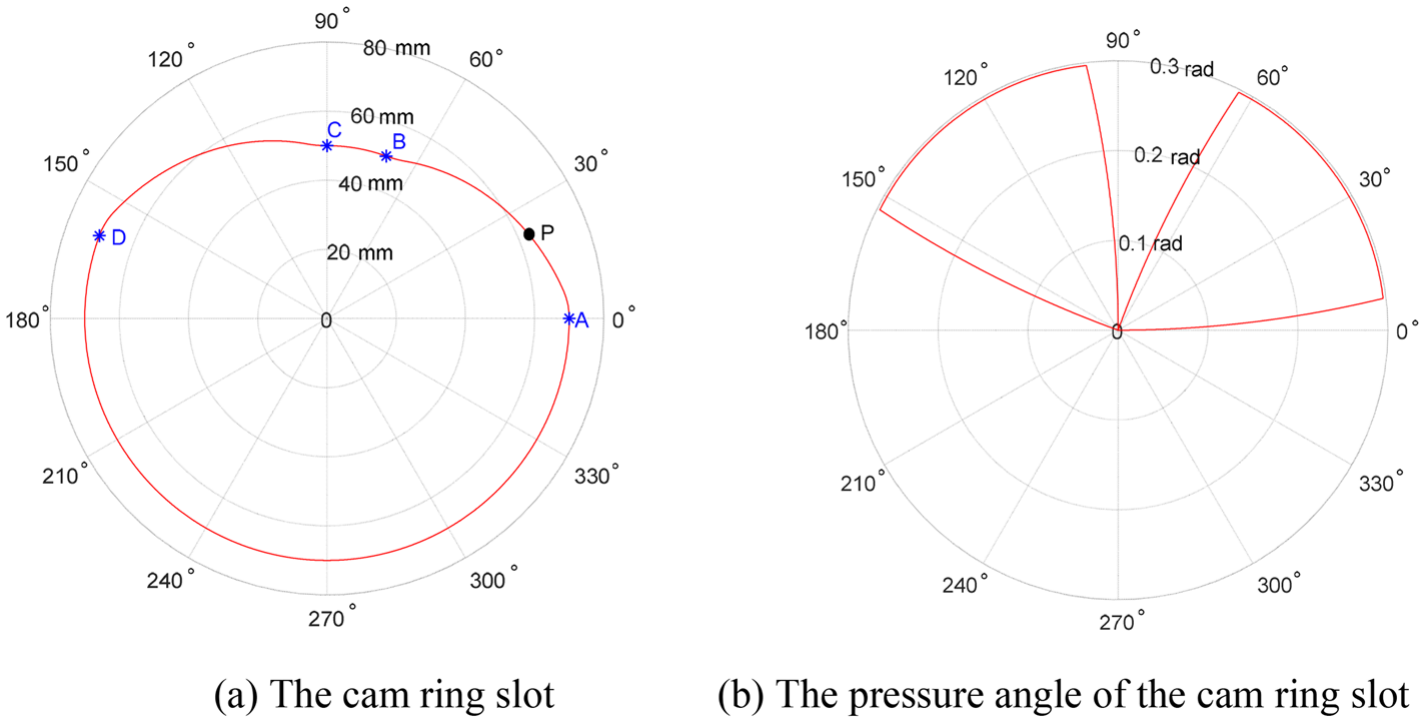
The calculated curve of the cam ring and its pressure angle. (Matlab2019a. https://ww2.mathworks.cn/products/get-matlab.html?s_tid=gn_getml).

Figure 6a is the cam ring slot of the prototype calculated with Eqs. (12) and (14). This cam slot curve is a closed curve composed of two concentric arcs (i.e., arc CB and arc DA) and two transition curves(i.e., curve CD and curve BA). The markers “*” are accurate positions of the aforementioned Point A, Point B, Point C, and Point D in Fig. 2, and “●” is the contact point between the sliding vane and the cam ring slot, which is named Point P and moves along the curve. Figure 6b presents the variation of the pressure angle when the sliding vane moves along the cam ring slot, which indicates the continuity of the pressure angle.

**Figure 7:**
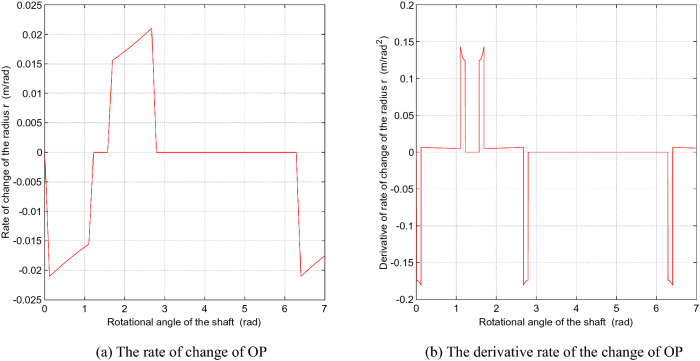
The change rate of the length OP and its derivative to the angle of the rotor shaft. (Matlab2019a. https://ww2.mathworks.cn/products/get-matlab.html?s_tid=gn_getml).

**Figure 8:**
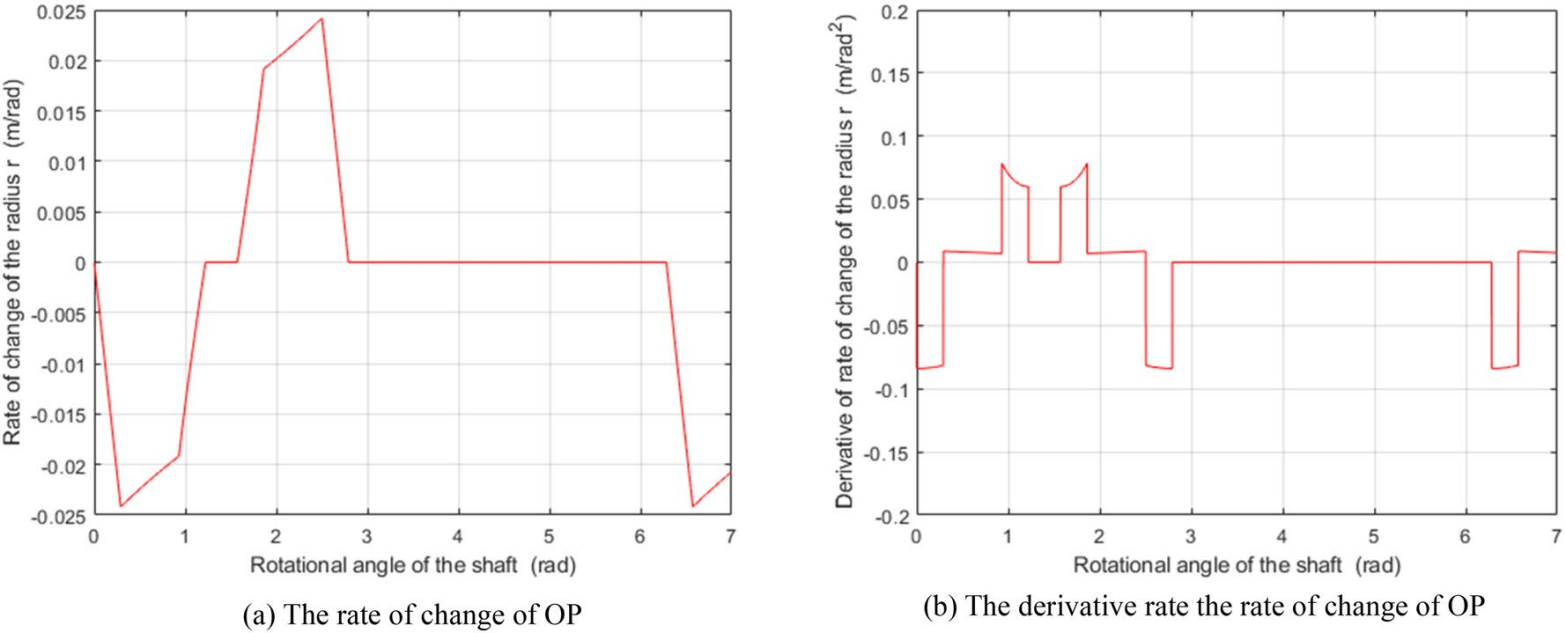
The rate of change of the length OP and its derivative when setting $\delta_{0}$ to ${\pi \mathord{\left/ {\vphantom {\pi 9}} \right. \kern-\nulldelimiterspace} 9}$. (Matlab2019a. https://ww2.mathworks.cn/products/get-matlab.html?s_tid=gn_getml).

Figure 7 shows the rate of change of the length OP and its derivative when the contact point P moves along the cam slot, where $r$ is the length of OP. The changing rate of the length OP is not a smooth curve, and the motions of the sliding vanes produce acceleration shocks acting on the shell. These shocks are stronger along with the higher rotational speed of the rotor shaft. Modifying the curve of the cam ring slot could minimize the shock. Figure 8 describes the rate of change of the length OP and its derivative when $\delta_{0}$ is ${\pi \mathord{\left/ {\vphantom {\pi 9}} \right. \kern-\nulldelimiterspace} 9}$. Compared with Fig. 7, the amplitude of the rate of change of the length OP increases about 0.004 m/rad, while the amplitude of its derivative decreases sharply to half of its previous value, which will relieve the acceleration shock.

### Calculation of fluid resistance coefficient

**Figure 9:**
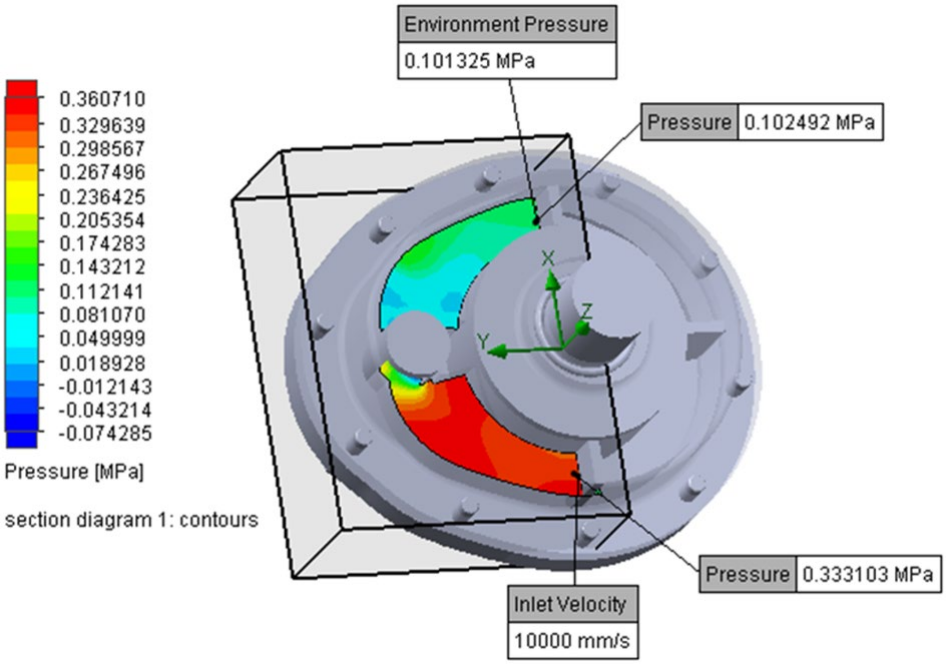
Boundary conditions and simulation result of the damper at certain valve angle. (FloEFD, trial version. https://www.plm.automation.siemens.com/global/en/products/simcenter/floefd.html).

The local resistance coefficient of the fluid is usually acquired with experiment or computational fluid dynamics (CFD) simulation. In this paper, a CFD software named FloEFD is adopted due to the lack of experimental conditions. FloEFD is a commercial software, which has the function of auto meshing and seamless integration with CAD software, e.g., Creo, CATIA, SolidWorks. In the simulation, water is the selected fluid, and Fig. 9 shows the boundary conditions and results of the simulation when the angle of the valve core is 26.08°. So, the local resistance coefficient of the fluid at this valve angle is acquired with Eq. (5).

In the prototype, the actual angle range of the valve core, $\theta_{{\text{V}}}$, is from zero, a full-open position, to 83.62°, a closed position. Because the simulation could not run when the valve core is in the closed position, the range in the simulation is from zero to a nearly closed-angle, 83.5°. Thus, a series of local fluid resistance coefficients are achieved according to Eq. (5) and the fluid pressures acquired from the simulation at each valve core position. The results in Table 2 validate the monotone increasing relationship between the local fluid resistance coefficients, $f{(}\theta_{{\text{V}}} {)}$, and $\theta_{{\text{V}}}$.

**Table 2:** the local fluid resistance coefficient at different valve core angle $\theta_{{\text{V}}}$.

$\theta_{{\text{V}}}$(°)	$f{(}\theta_{{\text{V}}} {)}$	$\theta_{{\text{V}}}$(°)	$f{(}\theta_{{\text{V}}} {)}$	$\theta_{{\text{V}}}$(°)	$f{(}\theta_{{\text{V}}} {)}$
0	1.79	45	22.00	78	967.00
5	1.86	50	24.82	79	1110.54
10	2.26	55	33.12	80	1417.39
15	3.25	60	49.51	81	5151.10
20	4.02	65	93.29	82	41,901.52
25	4.92	70	186.13	83	46,311.07
30	6.36	72	266.56	83.5	47,929.62
35	7.55	74	327.09		
40	10.33	76	453.64		

### Simulation and analysis of the damper

**Figure 10:**
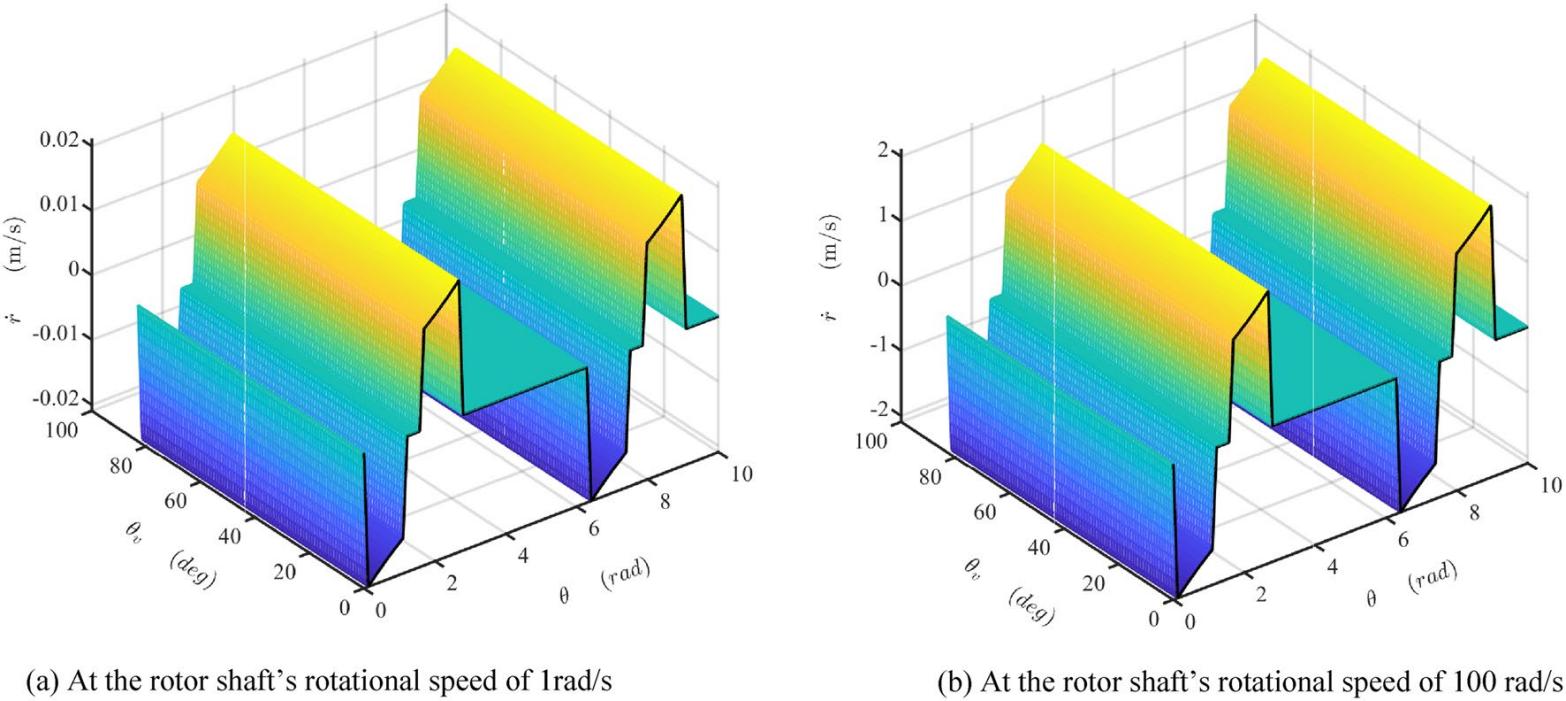
Radial speed map of the vane at two different the rotor shaft’s rotational speed. (Matlab2019a. https://ww2.mathworks.cn/products/get-matlab.html?s_tid=gn_getml).

**Figure 11:**
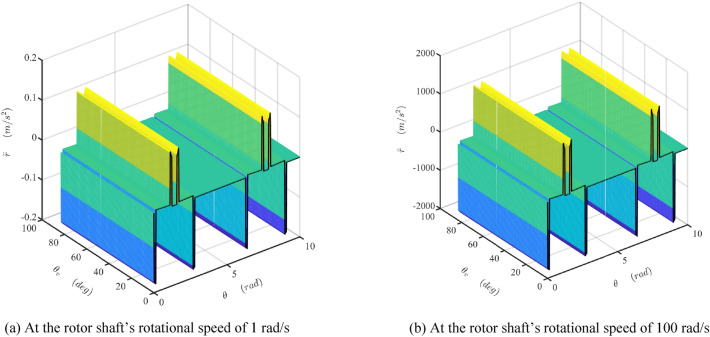
Extra radial acceleration map of the vane at two different the rotor shaft’s rotational speed. (Matlab2019a. https://ww2.mathworks.cn/products/get-matlab.html?s_tid=gn_getml).

**Figure 12:**
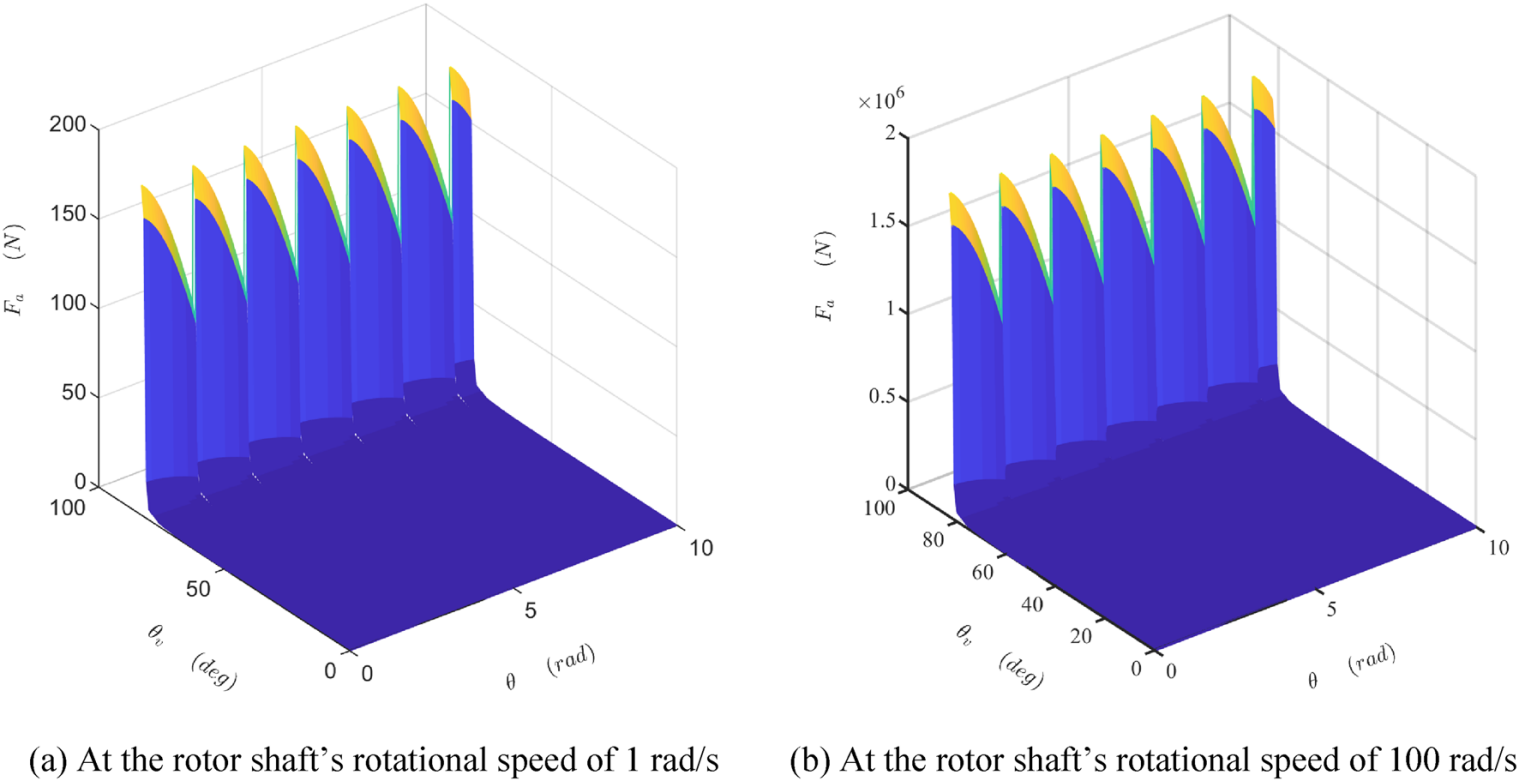
The map of the radial force produced by the fluid pressure acting on the cylinder surface of the rotor shaft at two different the rotor shaft’s rotational speed. (Matlab2019a. https://ww2.mathworks.cn/products/get-matlab.html?s_tid=gn_getml).

**Figure 13:**
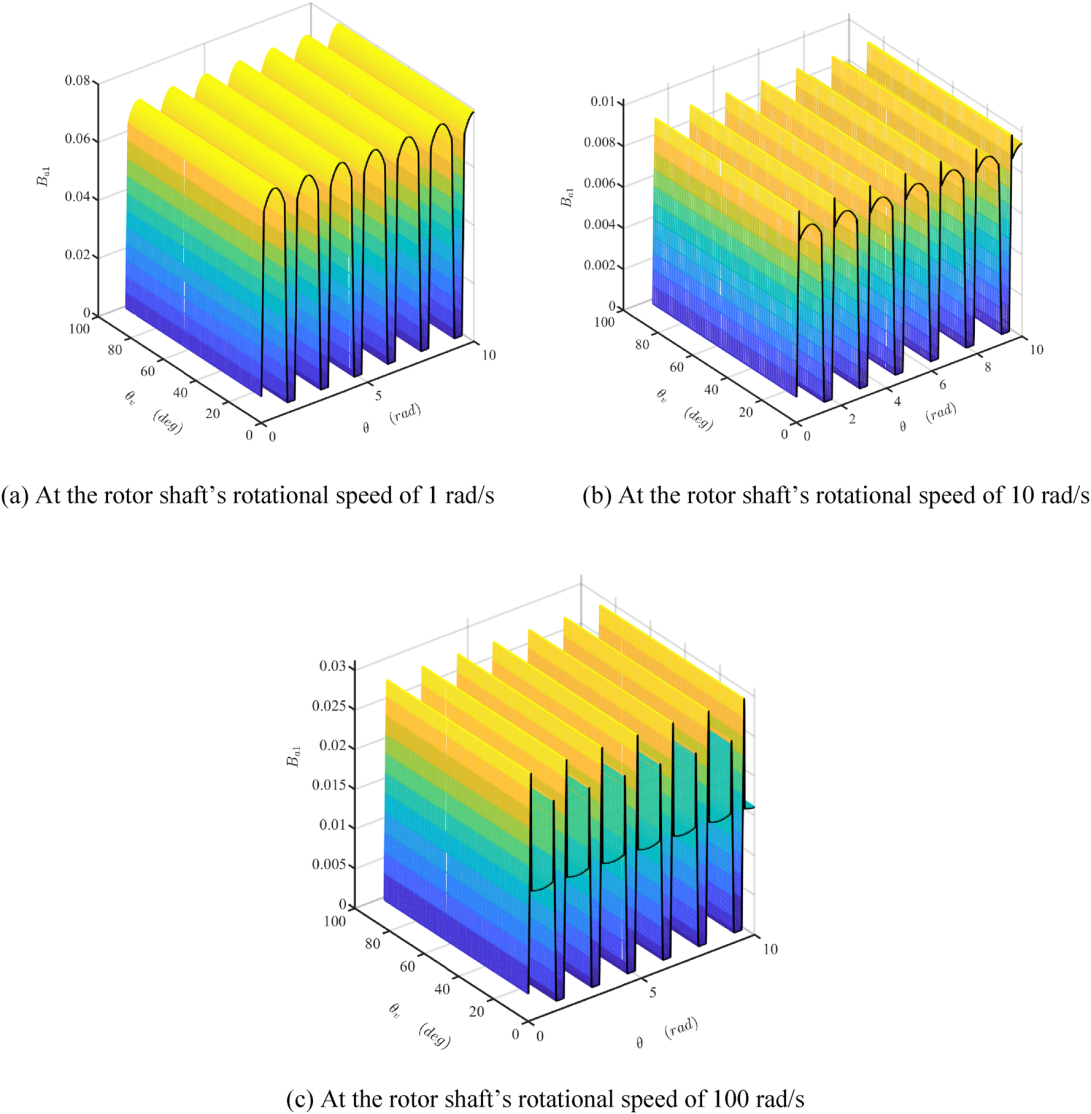
The map of the damper coefficient $B_{{{\text{a}}1}}$ at three different the rotor shaft’s rotational speeds. (Matlab2019a. https://ww2.mathworks.cn/products/get-matlab.html?s_tid=gn_getml).

**Figure 14:**
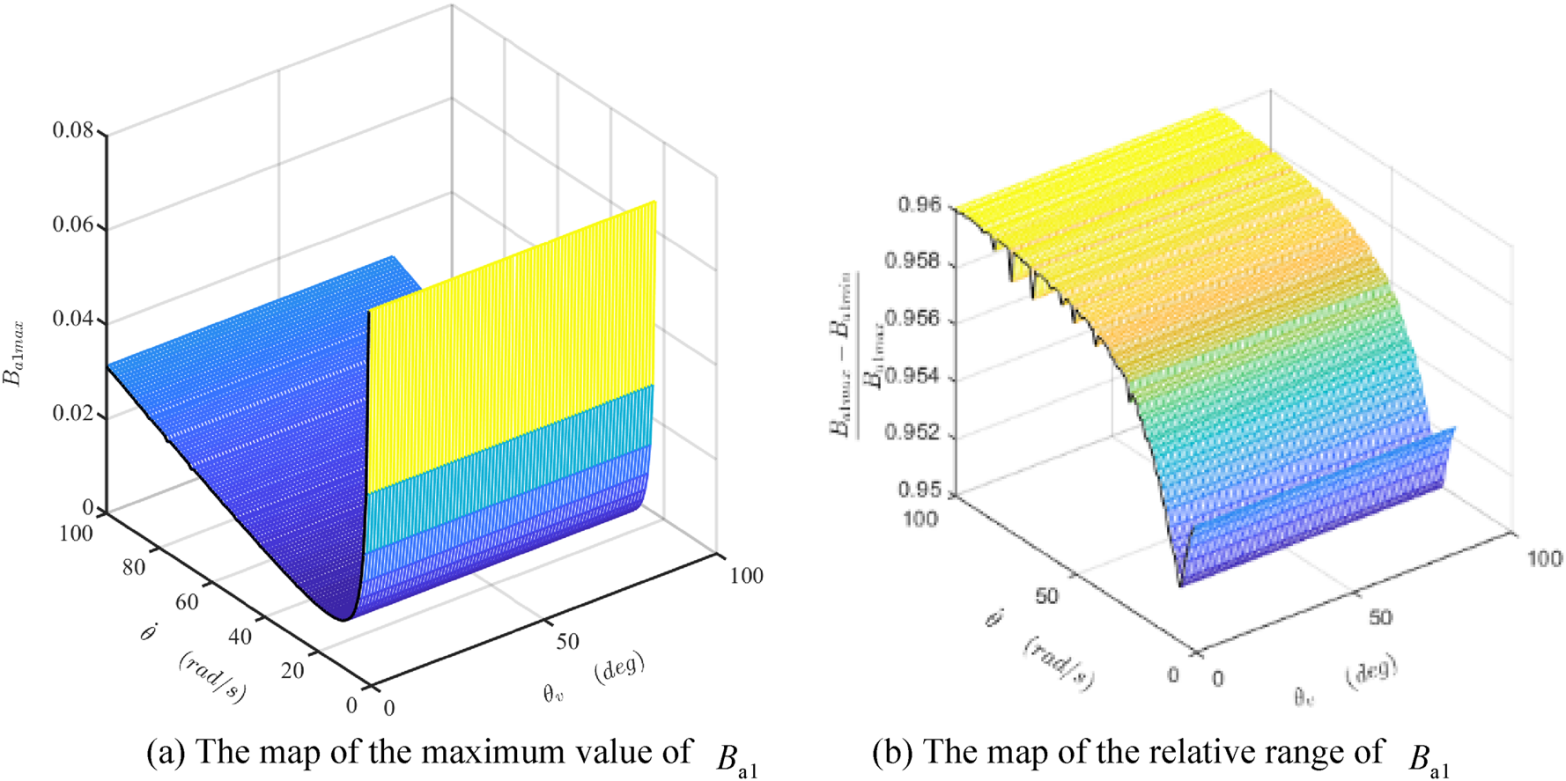
The maps of the maximum value and the relative range of the damper coefficient $B_{{{\text{a}}1}}$. (Matlab2019a. https://ww2.mathworks.cn/products/get-matlab.html?s_tid=gn_getml).

**Figure 15:**
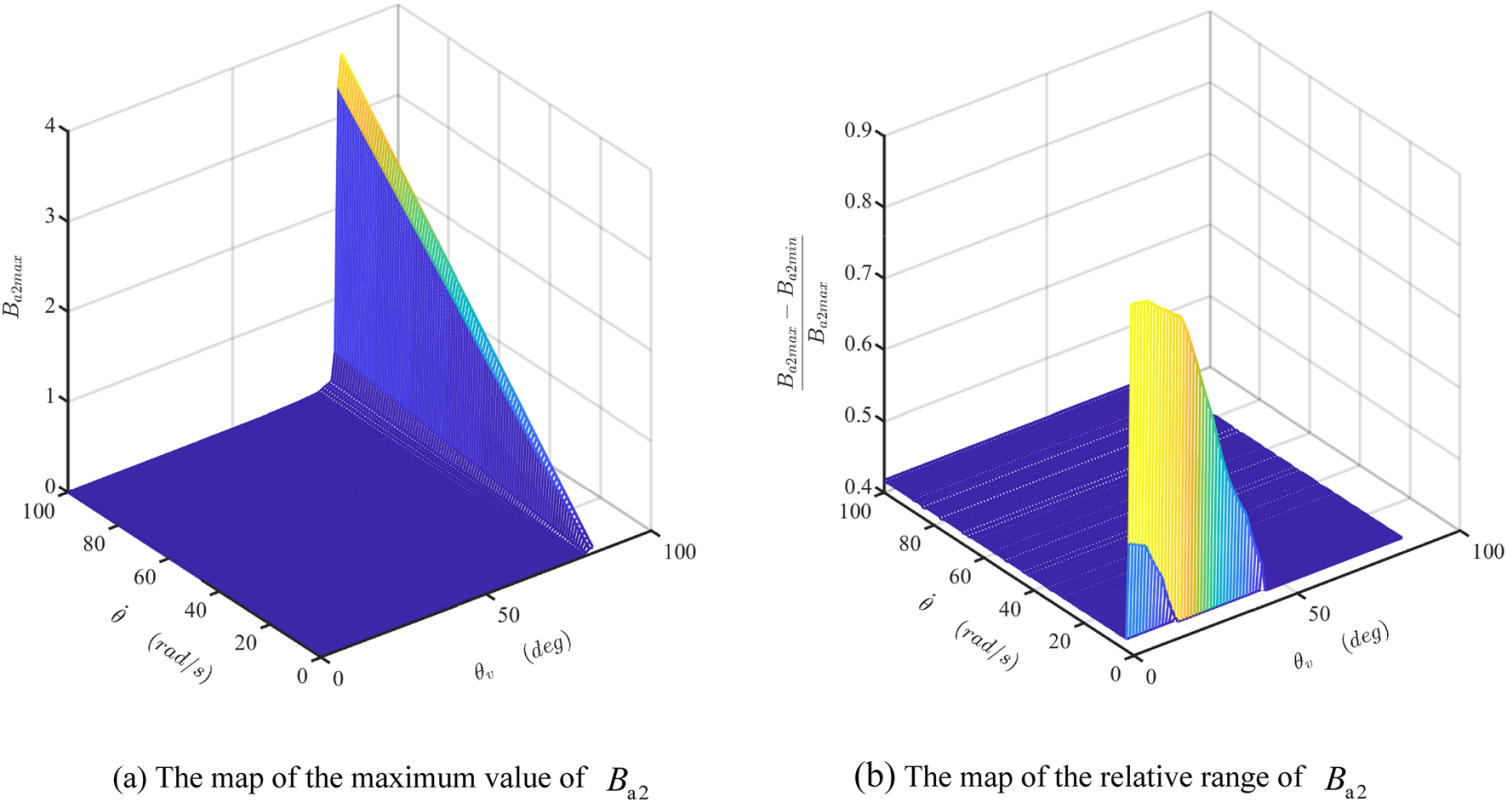
The maps of the maximum value and the relative range of the damper coefficient $B_{{{\text{a}}2}}$. (Matlab2019a. https://ww2.mathworks.cn/products/get-matlab.html?s_tid=gn_getml).

**Figure 16:**
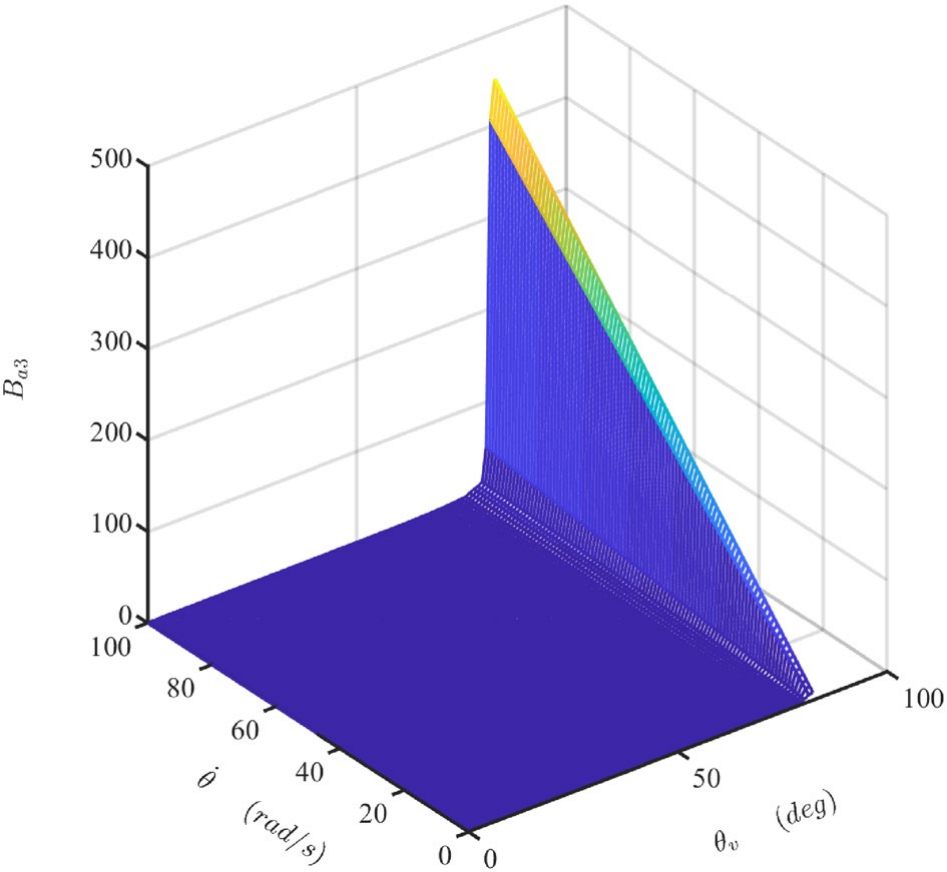
The map of the damper coefficient $B_{{{\text{a}}3}}$ (Matlab2019a. https://ww2.mathworks.cn/products/get-matlab.html?s_tid=gn_getml).

**Figure 17:**
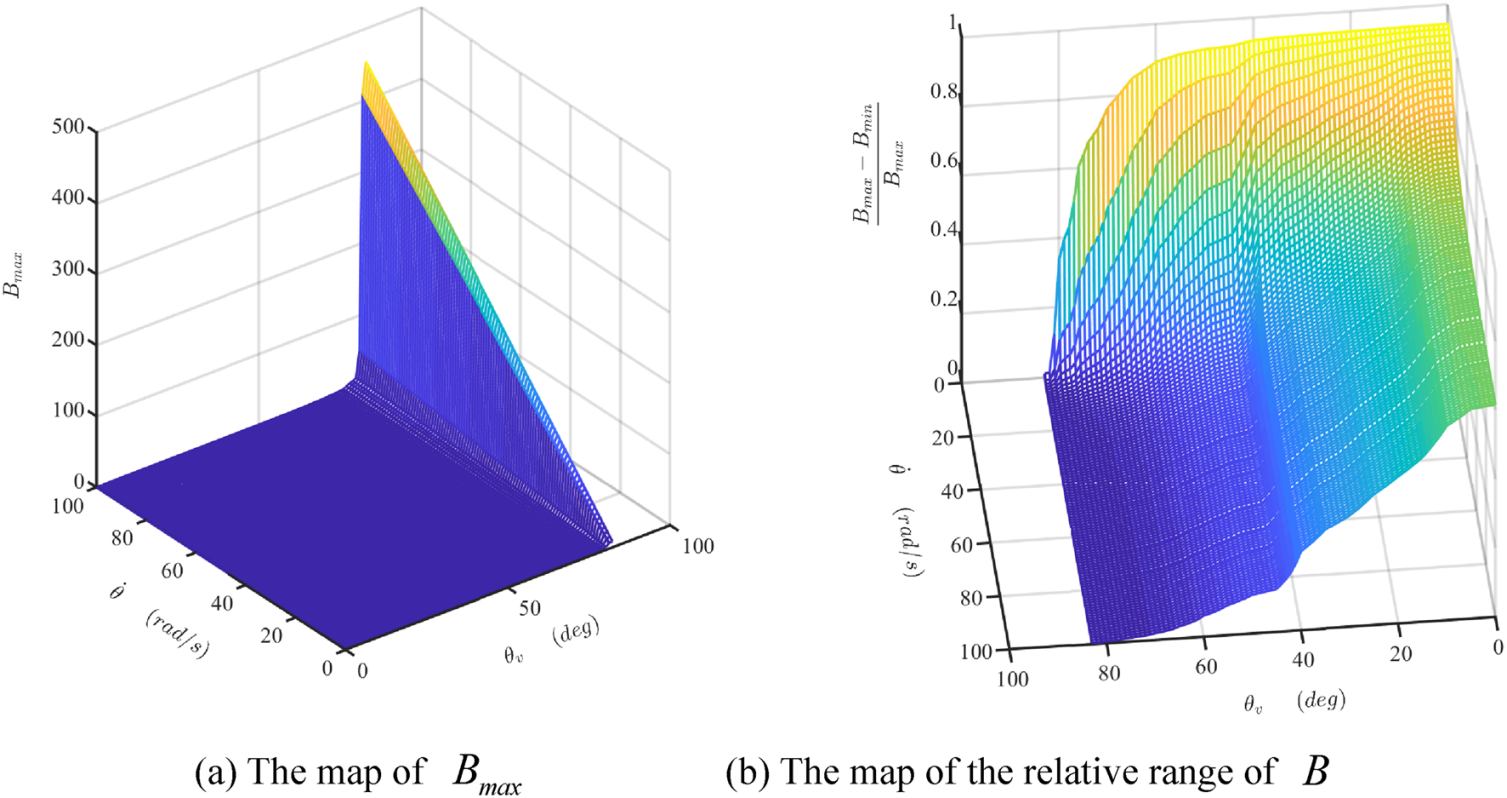
The maps of the maximum value and the relative range of the damper coefficient $B$. (Matlab2019a. https://ww2.mathworks.cn/products/get-matlab.html?s_tid=gn_getml).

**Figure 18:**
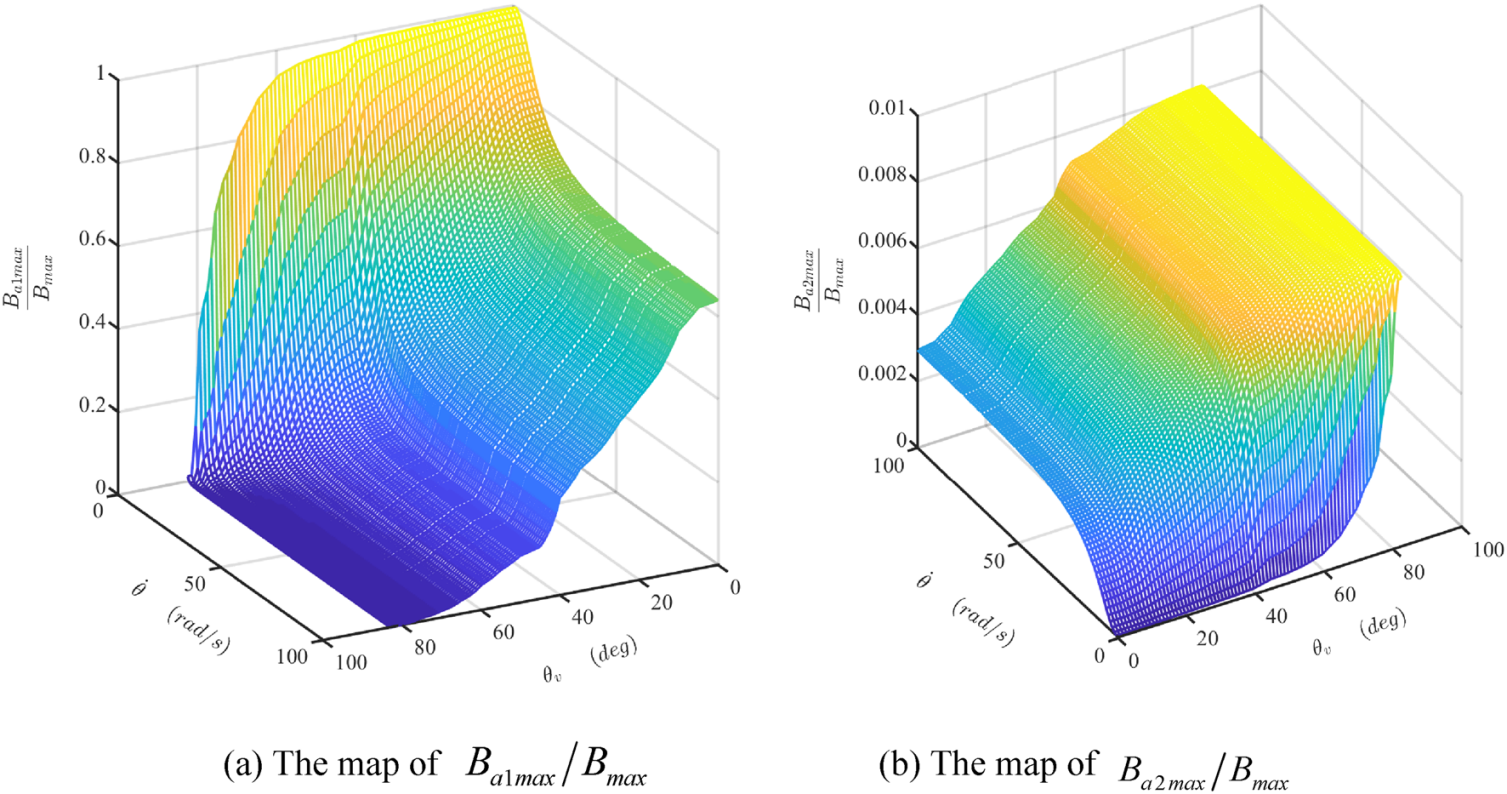
The maps of ${{B_{a1\max } } \mathord{\left/ {\vphantom {{B_{a1\max } } {B_{\max } }}} \right. \kern-\nulldelimiterspace} {B_{\max } }}$ and ${{B_{a2\max } } \mathord{\left/ {\vphantom {{B_{a2\max } } {B_{\max } }}} \right. \kern-\nulldelimiterspace} {B_{\max } }}$. (Matlab2019a. https://ww2.mathworks.cn/products/get-matlab.html?s_tid=gn_getml).

The damper's mathematical model is developed and simulated with Matlab/Simulink software (Matlab2019a. Available from https://ww2.mathworks.cn/products/get-matlab.html?s_tid=gn_getml), whose parameters are shown in Table 1. The motion of the sliding vane is analyzed, as shown in Figs. 10 and 11. The fluctuation of the radial force is detailed, as shown in Fig. 12, which is the fluid pressure drop acting on the side surface of the rotor. The fluctuation of the damping coefficient is discussed, as shown from Figs. 13, 14, 15, 16, 17 and 18, where the variables are the angular speed of the rotor shaft and the rotation angle of the valve core.

Figure 10a,b show the map of the radial velocity of the vane when the rotor shaft’s rotational speed is 1 rad/s and 100 rad/s, respectively. It is clear that the radial velocity of the sliding vane varies along with the shaft angle but is uninfluenced by the valve core's rotational angle. The black curve is the projection curve of the velocity surface in the $\theta_{{\text{V}}}$-axis direction, which is like the curve shown in Fig. 8a. The amplitude of the curve in Figs. 8a and 10a are equal, which is about one percent of that in Fig. 10b. It indicates that the amplitude of the vane's velocity map is proportional to the velocity of the rotor shaft.

Figure 11a,b show the map of the extra radial acceleration of the vane when the rotor shaft’s rotational speed is 1 rad/s and 100 rad/s, respectively. Similar to Fig. 10, the amplitude of the extra radial acceleration of the sliding vane varies along with the shaft angle but is uninfluenced by the angle of the valve core. The black curve is the projection curve of the acceleration surface in the $\theta_{{\text{V}}}$-axis direction, which is like the curve shown in Fig. 7b. The amplitude of the curve in Figs. 7b and 11a are equal, which is about 1/10,000 of that in Fig. 11b. It indicates that the amplitude of the radial acceleration of the vane is proportional to the square of the velocity of the rotor shaft. This extra acceleration will lead to the acceleration shock during the sliding vane moving along the transition curve guide, especially when the velocity of the rotor shaft is high. Modifying the transition curve could weaken this influence but could not eliminate it.

Figure 12 is the map of the force produced by the fluid pressure acting on the cylinder surface of the rotor shaft. Its amplitude increases with the increase of two parameters, the angle of the valve core and the rotational velocity of the shaft. But it fluctuates with the rotation of the shaft. Besides, this force enlarges the frictional resistance that the shell acting on the shaft and the corresponding frictional resistance torque. Therefore, the damper’s coefficient caused by this force is fluctuating and increasing. The shaft and the shell also suffer problems of strength, rigidity, or fatigue. A structure adopting symmetrically distributed valves can eliminate this problem but makes the damper larger and more complex.

Figure 13 presents the dynamic characteristic of the damper coefficient $B_{{{\text{a}}1}}$ at different shaft velocities. $B_{{{\text{a}}1}}$ is fluctuating along with the rotation of the shaft but is irrelevant to the rotating of the valve core. Comparing (a), (b), and (c) of Fig. 13, it is clear that the shape of $B_{{{\text{a}}1}}$ almost does not change in different rotational velocities of the shaft except a periodically superimposed pulse, whose amplitude increases with the increase of the rotational speed of the shaft. This pulse is produced by the extra radial acceleration, as shown in Fig. 11. The force that the transition curve guide acting on the sliding vane is bigger if the rotational velocity of the shaft or the extra radial acceleration is higher. Thus, the circumferential component of this force produces an extra resistance torque.

The combined action of the acceleration and friction torque makes the maximum value of $B_{{{\text{a}}1}}$ firstly decreasing and then increasing slowly with the increase of the rotating velocity of the shaft, as shown in Fig. 14a. This value is very small and could be neglected. From Fig. 14b, the relative range of $B_{{{\text{a}}1}}$ is bigger than 0.95, which means the fluctuation of $B_{{{\text{a}}1}}$ is very high.

Figure 15 indicates the maximum value of the $B_{{{\text{a}}2}}$ (denoted as $B_{{{\text{a}}2\max }}$) linearly increases in $\dot{\theta }$-axis and nonlinearly increased in $\theta_{v}$-axis. The relative range of $B_{{{\text{a}}2}}$ is about 0.42 in most areas. When both the shaft rotational velocity and the valve angle are small, the friction force acts as a dominant role, and $B_{{{\text{a}}2}}$ becomes very high sharply.

Figure 16 presents the map of $B_{{{\text{a}}3}}$ when the rotor shaft’s rotational speed increases from 1 to 100 rad/s. Similar to the tendency of $B_{{{\text{a}}2\max }}$, $B_{{{\text{a}}3}}$ increases linearly or nonlinearly with the increase of the shaft’s rotational velocity or the valve angle, respectively. But it does not vary with the rotation angle of the shaft. Comparing Figs. 14a, 15a, and 16, it is clear that $B_{{{\text{a}}3}}$ is a dominant component in the total damping coefficient when the rotational velocity of the shaft and the angle of the valve are big. Therefore, the maximum value of the damper coefficient, $B_{\max }$, is close to the $B_{{{\text{a}}3}}$, as shown in Fig. 17a. Consequently, the fluctuation of the total damping coefficient is smaller at the bigger value of the high rotational velocity of the shaft and the angle of the valve, as shown in Fig. 17b.

To analysis the influences of the components’ fluctuation on that of the total damping coefficient, a new index is adopted, which is the quotient of the maximum value of the component and that of the total damping coefficient. Figure 18a,b are the mesh surfaces of the indexes, i.e., ${{B_{a1\max } } \mathord{\left/ {\vphantom {{B_{a1\max } } {B_{\max } }}} \right. \kern-\nulldelimiterspace} {B_{\max } }}$ and ${{B_{a2\max } } \mathord{\left/ {\vphantom {{B_{a2\max } } {B_{\max } }}} \right. \kern-\nulldelimiterspace} {B_{\max } }}$, respectively. Figure 18a indicates that the fluctuation of $B_{a1}$ influences the damper coefficient $B$ greatly in regions of the low rotational velocity of the shaft and small angle of the valve. However, this influence could be ignored due to the small value of the damper coefficient component $B_{a1}$. Figure 18b suggests the maximum value of ${{B_{a2\max } } \mathord{\left/ {\vphantom {{B_{a2\max } } {B_{\max } }}} \right. \kern-\nulldelimiterspace} {B_{\max } }}$ is less than 0.01 m. The fluctuation of $B_{a2}$ has little influence on the damper coefficient $B$. Therefore, the fluctuation of the total damping coefficient decreases along with the increase of itself.

## Conclusion

This paper presents the structure and theoretical model of the concept of a novel rotational hydraulic damper, analyzes the performance of sliding vanes and characteristics of the damping coefficient with numerical simulation. Three conclusions are achieved from the simulation.

One is that the radial acceleration of the sliding vane varies periodically with the angular speed of the rotor, while whose amplitude is linear with the square of the rotor’s angular velocity. It increases sharply in the starting and the ending part of the transition curve during one period, which could cause the problem of the acceleration shock when the rotor’s rotational velocity is very high and limit the range of the damper’s angular velocity. Modifying the shape of the transition curve could ameliorate the problem.

The second is the nonlinearity of the damping coefficient. It contains two parts, fluctuation and non-linearly increasing with the rotational angle of the valve and the angular speed of the rotor. Dynamically adjusting the angle of the valve core could make the damping coefficient more linearly increase with the angular speed of the rotor. The fluctuation of the total damping coefficient is caused by two damping coefficient components, caused by the periodic radial motion of the sliding vanes and the periodic variation of the high-pressure fluid area, respectively. The former fluctuates violently while its amplitude is small. Its influence is bigger during the smaller value of the total damping coefficient. The latter’s relative fluctuant range is about 0.42 in most conditions, while its proportion is less than 0.01 in the total damping coefficient. Its influence on the total damping coefficient is negligible.

The last is that the sliding vanes have no radial motion when suffering pressure drop while almost do not suffer pressure drop during the radial motion, which relieves the worn of the sliding vanes. This structure and design idea could be implemented in the improvement of the rotational pump.

In further research, we will concentrate on improvements of the cam ring slot and the structure of the valve to smooth the radial acceleration curve and weaken the nonlinear relationship between the coefficient and the valve angle.

## Data Availability

The datasets supporting the conclusions of this article are included within the article.
